# Supplementary material for: Imaging-based indices combining disease severity and time from disease onset to predict COVID-19 mortality: A cohort study
Source: PLoS One. 2022 Jun 16;17(6):e0270111. doi: 10.1371/journal.pone.0270111 (PMC9202871; doi:10.1371/journal.pone.0270111)
Supplement: S1 Table — (DOCX) [file pone.0270111.s001.docx]

**S1 Table.** Models for death comparing CT-, CXR-, CRP-, and sO_2_- based measures of disease severity and severity/time indices.

|  | **Tested models for death** | | | | |
| --- | --- | --- | --- | --- | --- |
| **Severity measure** | **Base model (age and sex)** | **Base model + severity measure** | **Base model + severity measure + Charlson Comorbidity Index** | **Base model + severity measure + time from symptom onset** | **Base model + severity/time index** |
| **CT visual score** | | | | | |
| AIC | 249.49 | 232.22 | 236.54 | 230.05 | 231.16 |
| AUROC | 0.856 | 0.904 | 0.911 | 0.930 | 0.921 |
| **CXR RALE score** | | | | | |
| AIC | 249.49 | 237.50 | 242.11 | 234.98 | 240.72 |
| AUROC | 0.856 | 0.888 | 0.896 | 0.904 | 0.900 |
| **CRP** |  |  |  |  |  |
| AIC | 249.49 | 232.92 | 237.029 | 229.66 | 231.70 |
| AUROC | 0.856 | 0.868 | 0.880 | 0.887 | 0.879 |
| **sO_2_** |  |  |  |  |  |
| AIC | 249.49 | 249.15 | 252.54 | 245.44 | 246.90 |
| AUROC | 0.856 | 0.868 | 0.879 | 0.884 | 0.881 |

AIC, Akaike’s information criteria; AUROC, Area under the Receiver Operating Characteristic curve; CT, Computed tomography; CXR, chest X-rays; CRP, C-reactive protein; sO_2_, oxygen saturation level. AIC refers to Poisson models and AUROC to logistic models for death at 30 days.
