# Supplementary material for: Imaging-based indices combining disease severity and time from disease onset to predict COVID-19 mortality: A cohort study
Source: PLoS One. 2022 Jun 16;17(6):e0270111. doi: 10.1371/journal.pone.0270111 (PMC9202871; doi:10.1371/journal.pone.0270111)
Supplement: S2 Table — (DOCX) [file pone.0270111.s002.docx]

**S2 Table:** Characteristics associated with death in the validation cohort.

| Variables | All Patients | Deaths |  |
| --- | --- | --- | --- |
|  | **N (%)** | **N (%)** | **P*** |
|  | **215** | **48 (22.33)** |  |
| Age (years), median (IQR) | 63.8 (54.7-75.5) | 75.5 (66.3-81.1) | < 0.001** |
| Female sex | 67 (31.2) | 14 (29.2) | 0.735 |
| Days from symptom onset to CXR, median (IQR) | 6 (3-10) | 4.5 (2.5-7) | 0.005** |
| CXR RALE score, median (IQR) | 6 (2-13) | 8.5 (3-17) | 0.305** |

IQR, interquartile range; CXR, chest X-rays; RALE, Radiographic Assessment of Lung Edema. *Pearson's chi-squared test or Fisher exact test and p-value for the hypothesis of independence in the two-way table. ** P value nonparametric equality-of-medians test.
