# Supplementary material for: Imaging-based indices combining disease severity and time from disease onset to predict COVID-19 mortality: A cohort study
Source: PLoS One. 2022 Jun 16;17(6):e0270111. doi: 10.1371/journal.pone.0270111 (PMC9202871; doi:10.1371/journal.pone.0270111)
Supplement: S1 Fig — Receiver Operating Characteristic (ROC) curves of CXR RALE score (red line) and respective severity/time index (blue line) in multivariable logistic models for death at 30 days adjusted for age and sex. AUC values were: AUCCXR-RALE = 0.77 (95% CI, 0.71–0.84) and AUCCXR-RALE severity/time index = 0.79 (95% CI, 0.73–0.85). (DOCX) [file pone.0270111.s003.docx]

**S1 Fig. Validation cohort ROC curves.**


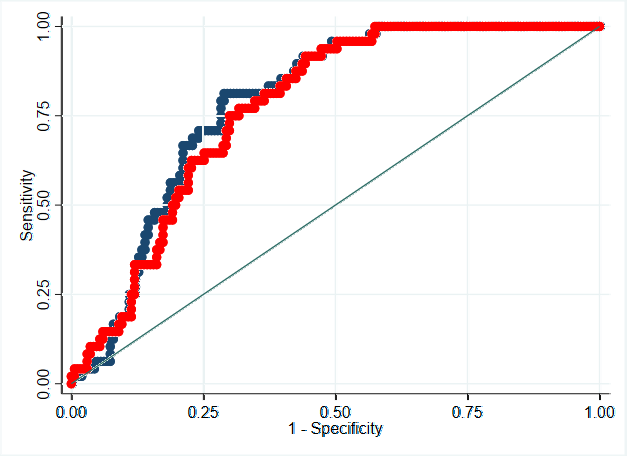


**S1 Fig.** Receiver Operating Characteristic (ROC) curves of CXR RALE score (red line) and respective severity/time index (blue line) in multivariable logistic models for death at 30 days adjusted for age and sex. AUC values were: AUCCXR-RALE = 0.77 (95% CI, 0.71-0.84) and AUCCXR-RALE severity/time index= 0.79 (95% CI, 0.73-0.85).
