# Supplementary material for: Imaging-based indices combining disease severity and time from disease onset to predict COVID-19 mortality: A cohort study
Source: PLoS One. 2022 Jun 16;17(6):e0270111. doi: 10.1371/journal.pone.0270111 (PMC9202871; doi:10.1371/journal.pone.0270111)
Supplement: Attachment — Submitted filename: reviewer comment PONE-D-21-29668 velocity index.docx [file pone.0270111.s004.docx]

PONE-D-21-29669 Reviewer comment

I would like to commend the authors for the time and dedication towards this manuscript. Technically, this is a good work however, I have some issues with the study design which need to be addressed.

1. **Aim of the study versus what was done**

Aim / Purpose of the study was stated as: “*The study aim was to evaluate indices of the velocity of disease progression to predict mortality of COVID-19 patients admitted to the emergency department (ED).”*

Your title and various sections in your manuscript referred to *“velocity of disease progression”.*

The term *“velocity of disease progression*” conferred the notion that, there were at least teo measurements of same severity measure within a time interval and then the velocity of progression computed from the change in severity measure over the time.

However, in your described methods, the time interval was measured from time of onset of symptoms to time of presentation at the emergency department, when the various tests were carried out. This measured interval directly measures, time from onset of symptoms to time of presentation at the emergency department which is influenced by various determinants including several factors contributing to delays in decision to seek care, delays in arriving at the facility and delays in the hospital before the measured tests were carried out. Please check this and revise you’re the aim and title of the study appropriately.

2. **Velocity indexes**

Also linked with the above comment, is a concern about the computation of the velocity index as described in lines 141- 147. “*In order to obtain indices that could indicate the velocity of disease progression before the first ED admission, we combined the CT visual score, the CXR RALE score, CRP levels, and sO2 levels separately with the time elapsed from symptom onset and the measurement of these parameters. The easiest way to obtain a velocity was to divide the severity measure by the time needed to reach that level of severity. This strategy was used for CRP levels and for CT- and CXR-based extension of parenchymal involvement, while sO2 velocity index was calculated as the difference in sO2 value to 100 (100-sO2) divided by the time from symptom onset to sO2 measurement.”*

This approach assumes that the baseline estimate for each severity measure was normal range and same value for all study participants, so that *“dividing the severity measure by the time needed to reach that level of severity”* would give a composite measure that is comparable among all study participants. However, based on past medical history of each study participants, the severity measure ie. sO_2_, CXR RALE, CT visual score, CRP at baseline which was not measured and practically difficult to determine based on the current study methodology, may be abnormal. This approach at estimating velocity index did not account for these variations in estimate at baseline. Please critically consider this.

3. **Interventions carried out**

There was no mention of the interventions at the emergency department which might have prevented outcome of death or delayed it beyond the 30days mark. I feel this is an important omission.

4. **Retrospective study or prospective study?**

Study design and selection of participants section, line 86 stated; “*This prospective cohort study included all consecutive patients aged > 18 years who presented to…..*”

And in same section, line 90 -91 stated that *“Given 91 the retrospective nature of the study, the Ethics Committee authorized the use of a patient’s data….”*

Please revise and be consistent

5. **Conclusion of the study**

Conclusion in abstract *“Indices describing the velocity of COVID-19 progression, especially those based on imaging, better predicted mortality than the same severity measures not incorporating the time needed to reach a certain level of severity*.

Conclusion as stated in the last paragraph of the discussion stated *“In conclusion, our study confirms that one of the most powerful prognostic factors for COVID-19 is the time from symptom onset to seeking medical assistance. This information is readily available and gives added value to the interpretation of other imaging and laboratory findings at ED presentation.”*

The conclusion in the abstract need to be revised to conform with what was stated in the last paragraph of the discussion

6. **Minor concerns**
a) Introduction, lines 48-49, “…more than 140 million cases and 3 million deaths have been reported globally up to May 2021 [1]” I will suggest a more current statistic.
b) Discussion section, lines 263-267 stated that *“The role of chest imaging has been widely evaluated, and even if the majority of available studies focus on CT scan [10-16], CXR-based scores have also been shown to be reliable in predicting COVID-19 outcomes [17-23]. This is particularly important since CT is not routinely recommended by the main international guidelines unless warranted by features of respiratory worsening, especially in resource-constrained environments, where CXR is more readily available [31]”*

This is true and the implication on your method of selection such that only patients with CT scan and Chest X-ray were included suggested that this study primarily admitted patients with features of worsening respiratory features. This limitation needs to be acknowledged.

Thank you
